# On geography and medical journalology: a study of the geographical distribution of articles published in a leading medical informatics journal between 1999 and 2004

**DOI:** 10.1186/1476-072X-4-7

**Published:** 2005-03-23

**Authors:** Maged N Kamel Boulos

**Affiliations:** 1 School for Health, University of Bath, Claverton Down, Bath BA2 7AY, UK

## Abstract

**Background:**

Studying the contribution of individual countries to leading journals in a given discipline can highlight which countries have the most impact on that discipline, and also give some idea about the geographical outreach of those journals. This paper examines the number of countries that contributed articles to one leading medical informatics journal, Medical Informatics & the Internet in Medicine, and the amount of their contributions between 1999 and the first half of 2004.

**Methods:**

The PubMed citations of all indexed articles from the chosen journal (n = 128) were retrieved online (up to Volume 29, Number 2/June 2004 issue, the latest indexed issue as at 28 January 2005). The country of corresponding author's affiliation for each retrieved citation was recorded. The five-year-and-half corpus of abstracts retrieved from PubMed was further explored using MetaCarta Geographic Text Search .

**Results:**

The examined journal has an international outreach, with authors from 24 countries, spanning four continents, contributing to the journal during the studied period. The journal is dominated by a very large number of articles from Europe (81.25% of all articles counted in this study), and in particular from the UK (15.63%) and Greece (15.63%). There were no contributions from Africa or South America.

**Conclusion:**

A detailed discussion and interpretation of these results and ideas for future analyses are provided. MetaCarta can prove very useful as a bibliometric research tool.

## Background

The study of the geographical distribution of journal publications as an indicator of the research output of individual countries has become a field of interest [1,2]. Studying the contribution of individual countries to leading journals in a given discipline can highlight which countries have the most impact on that discipline, and also give some idea about the geographical outreach of those journals.

Medical Informatics & the Internet in Medicine (MEDLINE Abbreviation: Med Inform Internet Med) is one of the most prestigious journals in the field of medical and health informatics, with a 2001 Impact Factor of 1.909 (ISI 2001 Journal Citation Reports-Science Edition – . In this paper, we examine the number of countries that contributed to Med Inform Internet Med and the amount of their contributions between 1999 and the first half of 2004.

We also demonstrate the use of MetaCarta Geographic Text Search appliance (GTS –  to sift through the corpus of Med Inform Internet Med abstracts from the same period through a geographical 'lens'.

Founded by a team of MIT researchers in 1999 with funding from the US CIA, MetaCarta bridges the gap between Geographical Information Systems (GIS) and text search, allowing users to link information to geography and to discover geographical themes within their documents. MetaCarta GTS scans documents and extracts geographical references, intelligently handling any geographical name ambiguities or inconsistencies it encounters. Users can search a geo-parsed document collection for the occurrence of any keywords (geographical or otherwise). GTS presents search results as icons on a map of the region of interest. The results are linked to the full documents. Users can further narrow searches by zooming in to specific geographical locations (the map acting as a filter), and by modifying their query string.

## Methods

The US National Library of Medicine PubMed service  was used to retrieve the PubMed citations of all indexed articles from Med Inform Internet Med as at 28 January 2005 (query string: 'Med Inform Internet Med [jour]'). The country of corresponding author's affiliation for each retrieved citation was recorded. If the affiliation of an entry could not be obtained from PubMed, it was looked up in the online version of the journal. A simple 'pen and paper' and Microsoft Excel analysis of the country of provenance of all retrieved citations was performed.

A MetaCarta GTS online demo account was created . The abstracts of all retrieved citations from PubMed were saved as individual HTML pages, one for each abstract. These were then compressed into a single zip archive and uploaded as a document collection to the MetaCarta demo account.

## Results

From 1999 (Volume 24, Number 1/March 1999 issue) to 2004 (Volume 29, Number 2/June 2004 issue) 128 articles from 24 countries were published in Med Inform Internet Med (Tables 1 and 2).

**Table 1:** Country contributions to Med Inform Internet Med Countries that contributed to Med Inform Internet Med and the amount of their contributions between 1999 and the first half of 2004.

**Country**	**Number of papers (1999 – June 2004)**
Australia	1
Cuba	1
Denmark	1
Hong Kong	1
India	1
New Zealand	1
Poland	1
Singapore	1
Slovenia	1
The Czech Republic	1
Taiwan	2
Israel	3
Finland	4
Austria	5
Spain	5
Sweden	5
USA	7
Japan	6
The Netherlands	6
France	7
Italy	13
Germany	15
Greece	20
UK	20

**Total: 24 countries**	**128 papers**

**Table 2:** Breakdown per year of Med Inform Internet Med papers and country contributions Breakdown per year of Med Inform Internet Med papers, contributing countries, and top countries and their individual contributions.

**Year**	**Number of papers**	**Number of Countries**	**Top countries (number of papers)**
1999	24	12	UK (6), Greece (4)
2000	20	9	Greece (5), UK (3)
2001	23	10	Germany (7), Greece (3), USA (3)
2002	24	14	UK (4), Greece (3), Italy (3)
2003	22	10	Greece (5), UK (4)
2004 (first 2 issues)	15	12	Austria (2), Italy (2), USA (2)

**Figure 1:**
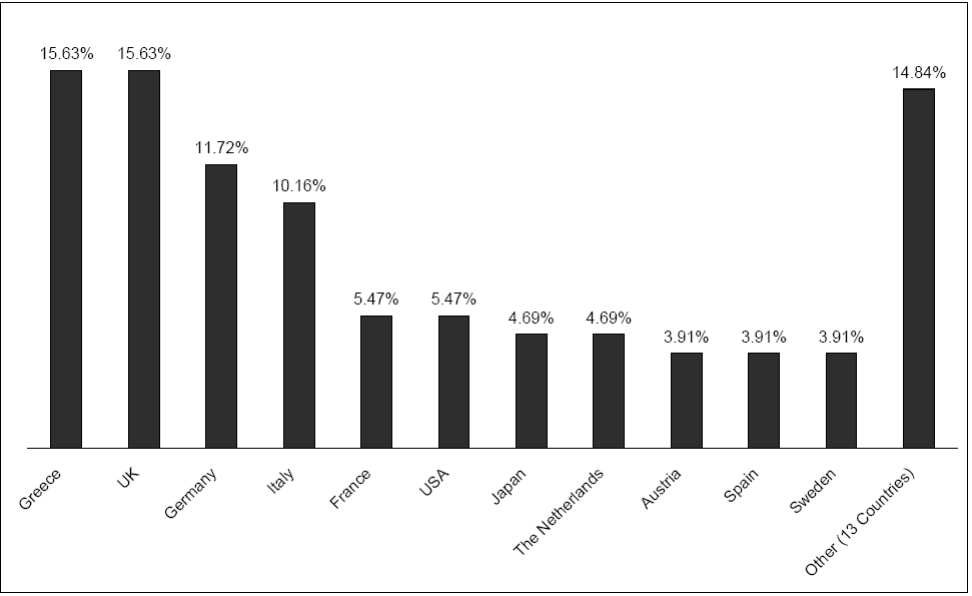
**Top contributing countries to Med Inform Internet Med and their share in per cent**. The leading countries in Med Inform Internet Med and their share in per cent during the period between 1999 and June 2004.

Authors from the UK contributed 15.63% and their colleagues from Greece another 15.63% of all articles published in Med Inform Internet Med during the period between 1999 and June 2004. Authors from Germany and from Italy were responsible for 11.72% and 10.16%, respectively, of all articles during the same period (Figure 1).

**Figure 2:**
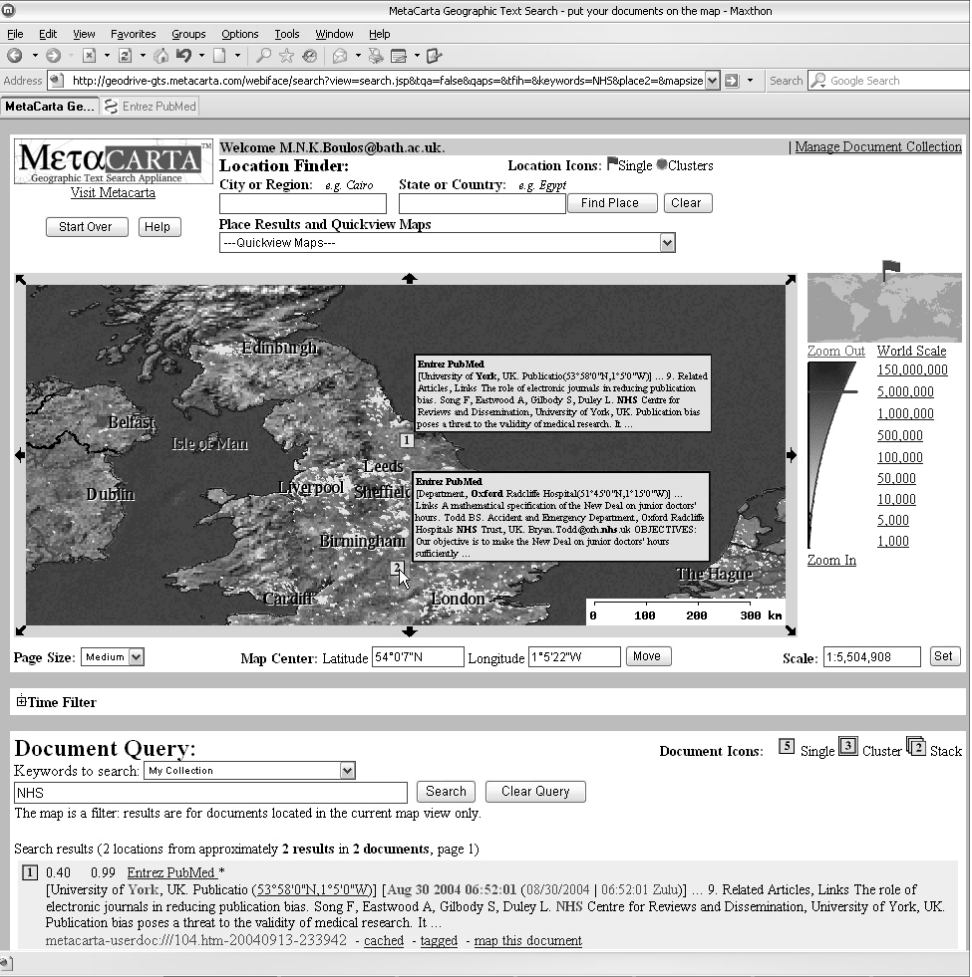
**Searching the corpus of Med Inform Internet Med abstracts (1999–June 2004) in MetaCarta**. Searching the corpus of Med Inform Internet Med abstracts (1999–June 2004) in MetaCarta for the keyword 'NHS' yielded two abstracts, one mapped to Oxford, UK, with two occurrences of the word 'NHS' in it (Med Inform Internet Med 2003 Jun, 28(2):129-34) and the other mapped to York, UK, with only one occurrence of the same word (Med Inform Internet Med 1999 Jul-Sep, 24(3):223-9).

The five-year-and-half corpus of Med Inform Internet Med abstracts retrieved from PubMed was further explored in MetaCarta by searching for the occurrence of different keywords in it and observing the results plotted on interactive geographical maps at various zoom levels. The search results are linked to the respective abstract pages, and the latter are usually linked to the full text of the articles on the publisher's Web site. Keywords can be:

• a geographical name, e.g., 'Amsterdam' (Med Inform Internet Med 2003 Sep, 28(3):209-19)

• an author's surname, e.g., 'Darmoni' (Med Inform Internet Med 2001 Jul-Sep, 26(3):165-78 and 2001 Oct-Dec, 26(4):325-30)

• the name of a project, e.g., 'Hip-Op' (Med Inform Internet Med 2002 Jun, 27(2):71–83 and 2003 Mar, 28(1):59–71)

• any other text string, e.g., 'electronic patient records' or 'NHS' (Figure 2), or

• a combination of any of the above to narrow searches.

## Discussion

Med Inform Internet Med has an international outreach, with authors from 24 countries, spanning four continents, contributing to the journal during the period between 1999 and June 2004, and contributions from at least nine countries to each yearly volume of the journal in the same period of time.

However, the journal is dominated by a very large number of articles from Europe (104 or 81.25% of all articles counted in this study), and particularly from the UK and Greece (which together account for 31.26% of all articles), with a small number of articles by comparison from the USA (5.47%), Japan (4.69%), Australia and the rest of the world (and none from Canada).

These findings might be partially explained by the fact that Med Inform Internet Med is a UK-based journal and also a European journal by geography (the UK being part of Europe). Previous results from other disciplines have also shown that a large proportion of papers originating from the UK appeared in British journals [1].

The observed geographical variation could be due to differences in the numbers of manuscript submissions from different countries and/or differences in rejection and acceptance rates for submitted manuscripts from different countries, with the possibility of some selection bias coming into play, e.g., reviewers (and perceived readership) preferring and evaluating more favourably papers from their own countries or some other country [1,3,4]. However, no data are available from Med Inform Internet Med to support or investigate such possibilities.

There were no contributions from any African or South American country during the same period. For Africa, this might be partially explained by the developing nature of the economies and infrastructures of the continent and the dampening effects this has on the research funding and productivity of African countries, and on their access to the Internet and the latest scientific periodicals. The 'brain drain' phenomenon might also be a contributory factor, with many African scientists and researchers being attracted to the West and conducting and publishing their research under the umbrella of Western institutions.

In South America similar factors operate [5,6], besides the fact that Spanish and Portuguese, rather than English, dominate the continent as the main spoken languages.

The theory of a bias against publications from the developing world might also partially explain the lack of contributions from Africa and South America [7]. It is noteworthy that Africa and South America were found to have the lowest number of biomedical publications per million population per year in a study by Rahman and Fukui published in 2003 [8].

### MetaCarta as a bibliometric research tool

Information resources and large textual datasets can be organised and navigated based on their geographical attributes. These geographical aspects of textual information are sometimes very useful as an index to information, providing an intuitive way of accessing, mining, and understanding it [9]. In these respects, MetaCarta can prove very useful as a bibliometric research tool. For example, by searching the relevant corpus of literature, MetaCarta can help identifying potential collaborators in one's research area of interest from one's same geographical region or across the world (geographically-aware information retrieval).

Metacarta geo-parses all geographical references in searched documents. For example, if an abstract by an author affiliated to a US institution is describing research that has been conducted in Germany and referring to related research conducted in Austria, then the abstract will be mapped to the three countries (USA, Germany and Austria). Although this could sometimes cause problems (if what one is looking for is to map or link each document to only one single location in some particular context), it could also prove a very powerful feature in other contexts.

### Ideas for future research

Other ideas for future and better analyses of this kind include:

• It is possible for some articles resulting from international cooperation to have authors from more than one country, but this study only considered the country of corresponding author's affiliation. It would be useful to consider this possibility in future studies. PubMed and MEDLINE only include the corresponding author's institutional affiliation and address, so the actual journal articles will have to be checked for the details of other authors.

• Comparing the geographical distribution of the readership of Med Inform Internet Med (using data from subscriptions to the printed edition and the access logs of the online journal articles) and that of the articles it publishes might help interpreting some of the geographical variation in the latter, since authors usually select journals they can access and read or are at least aware of to publish their papers in.

• Smaller affluent nations like Belgium, Finland, Israel and Sweden often have higher publication activity rates than larger countries like Germany, the UK and the USA, but this is not readily apparent when looking at the gross or absolute publication productivity numbers of the different countries. A good way around this is to normalise publication productivity data to population size or GDP of respective countries (publications per million inhabitants/year or publications per billion US dollars GDP/year) [10-13].

• It would be interesting to repeat the same study after five years and compare the results. Reference [14] provides a related example.

• It would also be useful to see how Med Inform Internet Med compares to other leading and newer journals in the field of medical and health informatics regarding the geographical distribution of their published articles, and to see if there are any common trends across journals.

• Also, breaking down the geographical distribution of articles published in a given journal or collection of journals by article/study type and by research topic/theme over the studied period of time could help revealing how medical and health informatics thinking and applications are evolving worldwide, as well as any regional themes. Reference [15] provides a related example.

• Tracking multiple articles by the same authors and institutions covering same, similar or different research topics or projects could also prove useful in identifying evolving research trends, centres of expertise, existing collaboration nuclei (and potential collaborators) in different research areas from around the world [16].

• Studying the geographical distribution of editorial board members of leading medical and health informatics journals and relating the findings to the geographical distribution of articles published in the same journals could yield additional useful insight. References [17] and [18] provide two related examples.

## Conclusion

This paper has provided a detailed discussion and interpretation of the results of a study of the geographical distribution of articles published in a leading medical informatics journal between 1999 and 2004. Studying the contribution of individual countries to leading journals in a given discipline can highlight which countries have the most impact on that discipline, and also give some idea about the geographical outreach of those journals. Ideas for future analyses were also presented. MetaCarta can prove very useful as a bibliometric research tool.
